# Advancing Myocardial T1 Mapping: A Comparative Study of the Frequency‐Independent MFA Sequence and Standard MOLLI

**DOI:** 10.1002/nbm.70031

**Published:** 2025-04-02

**Authors:** Vitali Koch, Mukaram Rana, Christina Seppi, Simon Martin, Thomas Vogl, David M. Leistner, Marco M. Ochs, Sebastian M. Haberkorn

**Affiliations:** ^1^ University Hospital Department of Radiology Goethe University Frankfurt Frankfurt Germany; ^2^ University Hospital Department of Cardiology and Angiology Goethe University Frankfurt Frankfurt Germany; ^3^ Teaching Hospital of Frankfurt Medical School Department of Gynecology Kettler Medical‐Centre Offenbach Germany; ^4^ German Center of Cardiovascular Research (DZHK) Frankfurt Germany; ^5^ Cardiopulmonary Institute (CPI) Frankfurt Germany

**Keywords:** cardiac MRI, heart rate independence, MOLLI, multiflip angle, myocardial relaxation times, myocardial tissue characterization, quantitative imaging, T1 mapping

## Abstract

T1 mapping is essential for detecting myocardial changes, but standard methods like the MOLLI sequence are limited by heart rate dependency and sensitivity to motion artifacts. This study introduces the multiflip angle (MFA) sequence as a novel alternative, aiming to provide frequency‐independent and robust T1 mapping, particularly in challenging cardiac conditions. The novel MFA sequence was validated using nickel (II) chloride phantoms and systematically compared with the standard MOLLI sequence in 20 healthy volunteers using a 1.5 Tesla Philips Achieva MRI system. T1 values were assessed at rest and under mild physical exertion to evaluate frequency dependency, measurement precision, and robustness to motion artifacts. The MFA sequence demonstrated robust frequency independence, with T1 values remaining stable across varying heart rates, unlike MOLLI, which exhibited a significant correlation between T1 values and heart rate (*R* = 0.52, *p* < 0.001), and sex (3% higher values in females; *p* = 0.044). Although both sequences showed no statistically significant age‐related differences, MOLLI yielded more precise T1 measurements with lower variability compared to MFA. Additionally, MFA exhibited reduced susceptibility to motion artifacts, maintaining consistent values across myocardial regions and physiological conditions, particularly in basal segments where MOLLI showed greater variability. The MFA sequence offers a frequency‐independent and motion‐robust alternative to the MOLLI sequence for myocardial T1 mapping. Although the MOLLI sequence provides higher precision, MFA's stability across varying heart rates and resistance to motion artifacts positions it as a promising option, particularly for patients with arrhythmias or during stress testing. Further investigation is warranted to refine its clinical applications.

## Graphical abstract

The multiflip angle (MFA) sequence provides a frequency‐independent and motion‐robust alternative to the MOLLI sequence for myocardial T1 mapping. Although MOLLI demonstrated higher measurement precision, it showed heart rate dependency and sex‐related differences in T1 values. In contrast, MFA maintained stable T1 values across varying heart rates and exhibited reduced susceptibility to motion artifacts. These findings highlight MFA's potential for improved myocardial characterization, particularly in patients with arrhythmias or during stress testing, warranting further clinical evaluation.

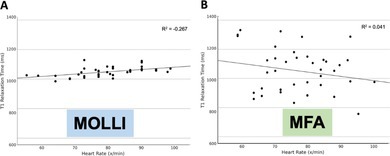

**Abbreviations**
CMR: cardiac magnetic resonance
FLASH: fast low angle shot
FOV: field of view
ICC: intraclass correlation coefficient
MFA: multiflip angle
MOLLI: modified Look–Locker inversion recovery
NiCl_2_
: nickel (II) chloride
SASHA: saturation recovery single‐shot acquisition
shMOLLI: shortened MOLLI

## 1 Introduction

Cardiovascular magnetic resonance (CMR) imaging has become an essential diagnostic tool in cardiology, enabling the assessment of cardiac diseases, structures, and functional impairments with high spatial resolution and excellent soft‐tissue contrast, all without ionizing radiation [1, 2]. T1 mapping, a noninvasive method to detect myocardial changes, provides a visual representation of myocardial T1 relaxation times using color‐coded maps. The modified Look–Locker inversion recovery (MOLLI) sequence, introduced in 2004, has become the standard for myocardial T1 mapping [3, 4]. Despite its widespread use, MOLLI has notable limitations, particularly its dependence on heart rate and susceptibility to errors in patients with arrhythmias, such as atrial fibrillation or tachycardia [3, 5, 6]. This heart rate dependency can lead to inaccurate T1 measurements, affecting clinical decision‐making [6, 7].

In response to these limitations, alternative T1 mapping sequences have been developed, including the Shortened MOLLI (shMOLLI) and Saturation Recovery Single‐Shot Acquisition (SASHA) [8]. However, these alternatives often exhibit reduced precision compared to MOLLI [9]. Consequently, there is a need for a T1 mapping sequence that maintains accuracy while being independent of heart rate and less affected by arrhythmias.

The multiflip angle (MFA) sequence represents a novel approach to myocardial T1 mapping. Unlike MOLLI, which uses a single flip angle, the MFA sequence applies multiple, variable flip angles, improving spatial resolution and creating a detailed three‐dimensional (3D) T1 map of the myocardium [10, 11]. The MFA sequence employs a gradient‐echo technique known as fast low angle shot (FLASH), which minimizes the repetition time and stabilizes the imaging process. This “spoiled” gradient‐echo technique intentionally destroys remaining transverse magnetization, allowing only the longitudinal magnetization to contribute to the T1‐ or T2‐weighted image [12, 13]. The MFA approach incorporates a DESPOT1 algorithm, which calculates T1 values from several spoiled gradient‐echo images obtained at different flip angles [14, 15]. Preliminary studies, such as those by Coolen et al. [13], have shown promising results with the MFA sequence, demonstrating its capability to generate consistent T1 maps even at high heart rates in mouse models.

The primary goal of this study is to develop and validate the MFA sequence as an alternative to the standard MOLLI sequence. We aim to test whether the MFA sequence can overcome MOLLI's limitations, specifically its heart rate and arrhythmia dependence. This study includes validation through phantom measurements, followed by in vivo testing on human subjects. Comparisons between the MFA and MOLLI sequences are made under both rest and stress conditions to determine heart rate effects on T1 values, with each subject undergoing measurements with both sequences.

By investigating whether the MFA sequence provides more stable T1 measurements across different heart rates and arrhythmia conditions, this study seeks to establish a more robust, accurate method for myocardial T1 mapping. The findings have potential implications for clinical practice, especially in providing reliable T1 values for patients with variable heart rates, ultimately leading to improved diagnostic and therapeutic strategies in cardiovascular care.

## 2 Methods

This study aimed to standardize the novel MFA sequence in a controlled experimental setting and to systematically compare it with the conventional MOLLI sequence for myocardial T1 mapping. Ethical approval was obtained from the medical faculty's ethics committee (Study No. 2020‐844) following the principles of the Declaration of Helsinki.

### 2.1 Study Cohort

Twenty healthy volunteers (10 males and 10 females), aged 22 to 31 years, were recruited for this study through a flyer campaign at the university. A power analysis using G*Power indicated that a sample size of 20 was sufficient for statistical significance in a paired study design [16].

- Inclusion criteria:
  ○ Written informed consent
  ○ Age between 18 and 35 years
  ○ No known cardiovascular or chronic illness
- Exclusion criteria:
  ○ Structural heart disease
  ○ Symptoms indicative of heart disease
  ○ MRI contraindications (e.g., pacemakers and metal implants)
  ○ Pregnancy

### 2.2 Study Design

The study protocol included two measurement phases for each participant: at rest and under mild physical exertion. Resting heart rate was first recorded, followed by T1 measurements using both the MOLLI and MFA sequences. To achieve a controlled increase in heart rate without pharmacological intervention, participants performed repeated squeezing exercises with an MRI‐compatible stress ball. After each set of 100 repetitions, heart rate was measured again, and once it had increased by at least 10% from baseline, imaging was repeated under these stress conditions. This setup allowed a systematic comparison of T1 values between the MFA and MOLLI sequences under varying physiological conditions [17].

### 2.3 Validation of MFA Sequence

Prior to in vivo imaging, the MFA sequence was validated using an experimental phantom composed of nickel (II) chloride (NiCl_2_) solutions with predetermined T1 relaxation times [18, 19]. NiCl_2_ provides stable magnetic susceptibility across a broad concentration range, making it an ideal standard for T1 mapping calibration. Phantoms with NiCl_2_ concentrations ranging from 0.25 to 13.0 mM were used to test the accuracy of T1 measurements generated by the MFA sequence in a controlled environment [20, 21].

### 2.4 Imaging Protocol

All MRI scans were performed on a 1.5 Tesla MRI system (Philips Achieva, Netherlands) equipped for cardiac imaging.

- MOLLI sequence specifications: The MOLLI protocol employed a 5(3)3 acquisition scheme, with the following parameters: field of view (FOV) 256 × 256 mm, echo time (TE) 1 ms, repetition time (RT) 0.6 ms, flip angle 35°, and slice thickness 8 mm. The scan duration per sequence was approximately 16 heartbeats [22, 23].
- MFA sequence specifications: The MFA sequence used multiple flip angles (2°, 5°, 8°, 11°, and 14°) to generate homogenous T1 mapping. Key parameters included FOV 256 × 256 mm, TE 1.25 ms, RT 2.5 ms, and slice thickness 8 mm. Total scan time for each MFA sequence was approximately 12 min [24].

### 2.5 Data Analysis

**FIGURE 1:**
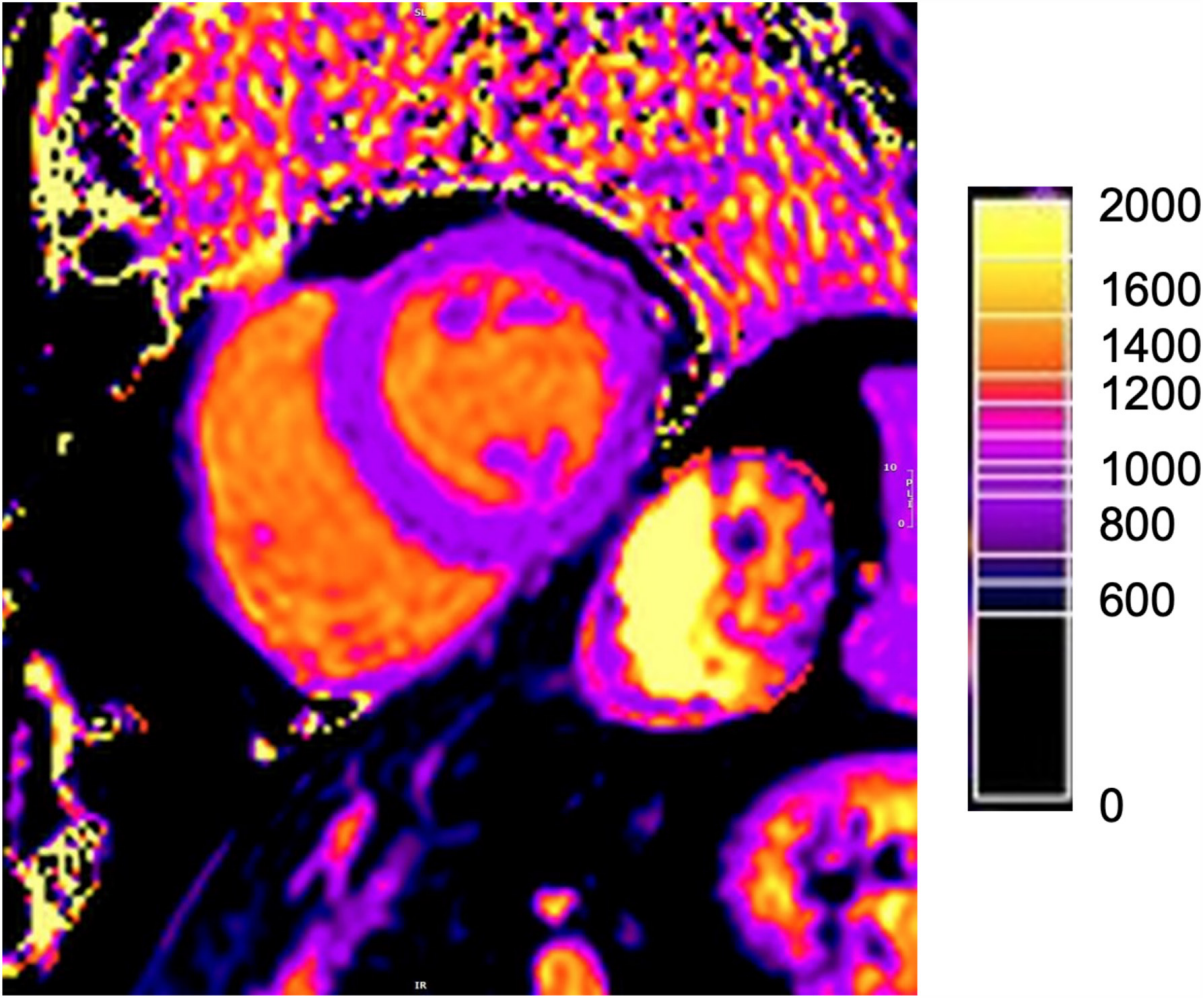
MOLLI sequence with a color‐coded T1 map, displaying the legend for T1 relaxation times in milliseconds and their respective color coding in the upper left corner of the image.

**FIGURE 2:**
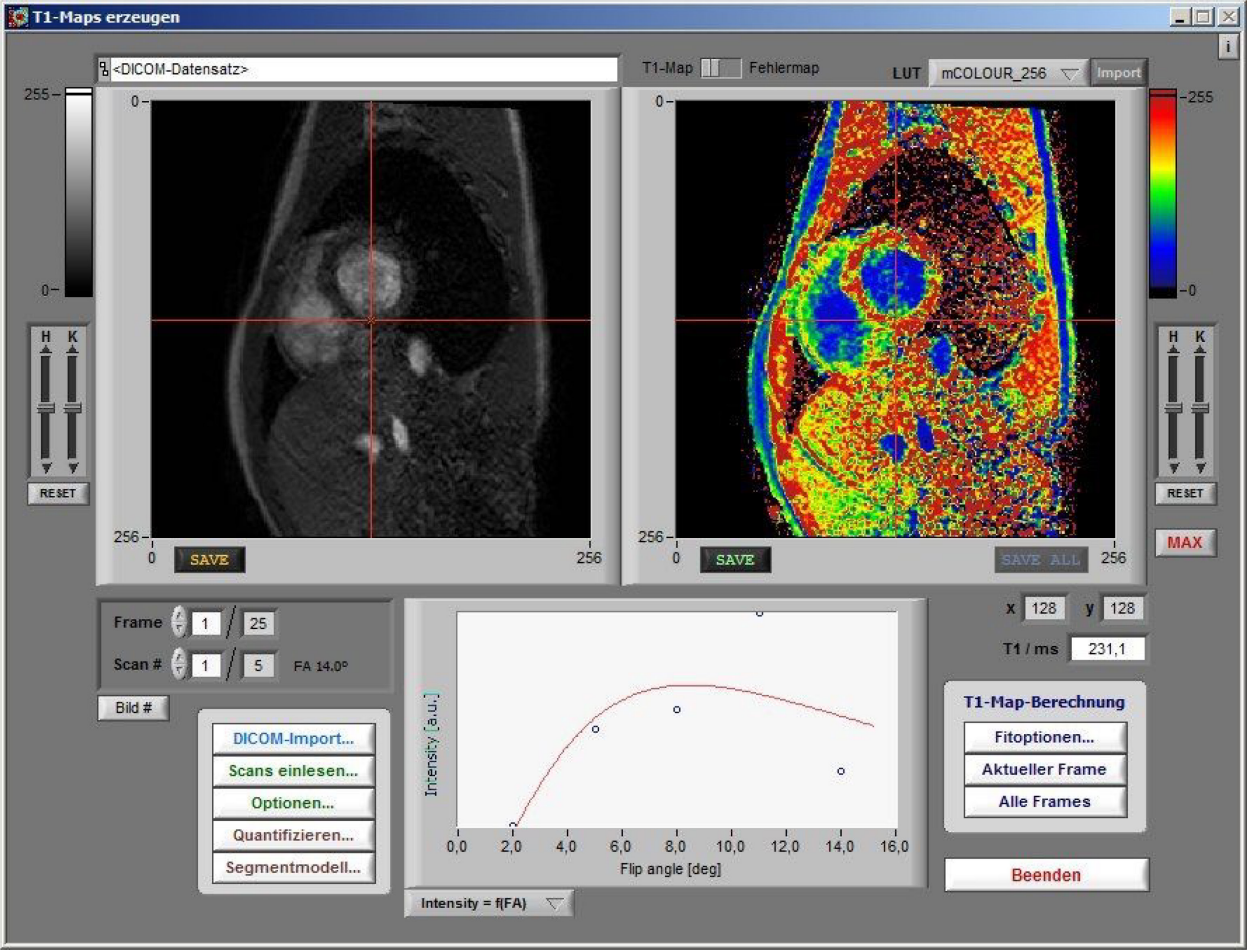
T1 mapping with the MFA sequence: The native image is shown in the top left, the color‐coded T1 relaxation times on the right, and the corresponding flip angles displayed at the bottom.

Two independent observers analyzed the T1 maps from both the MOLLI and MFA sequences (see Figures 1 and 2). T1 relaxation times were measured globally for the left ventricle and across 16 standardized myocardial segments, with both resting and stress‐induced images included in the analysis. MOLLI data were processed using Circle Cardiovascular Imaging software, whereas MFA sequence data were analyzed using a custom LabView‐based program developed by the research team (see Figure 2) [24].

### 2.6 Statistical Analysis

Data analysis was conducted using IBM SPSS Version 29. Continuous variables are presented as mean ± standard deviation. Paired *t* tests were used to compare resting and stress‐induced T1 values within subjects. The Pearson correlation coefficient was employed to examine relationships between T1 values and variables such as age and heart rate. Interobserver variability was assessed using the intraclass correlation coefficient (ICC), and Bland–Altman plots were generated to visualize measurement agreement between observers. Statistical significance was set at *p* < 0.05.

This comprehensive setup and analysis provide a robust foundation for evaluating whether the MFA sequence can yield stable T1 measurements across various heart rates, potentially establishing it as a viable alternative to MOLLI in both clinical and research applications.

## 3 Results

The validation and application of the MFA sequence for myocardial T1 mapping demonstrated its frequency independence and stability compared to the MOLLI sequence. This section summarizes the findings, focusing on the superior reliability of MFA, particularly regarding its frequency and motion independence, as well as its lower result variability.

### 3.1 Phantom Validation of the MFA Sequence

**FIGURE 3:**
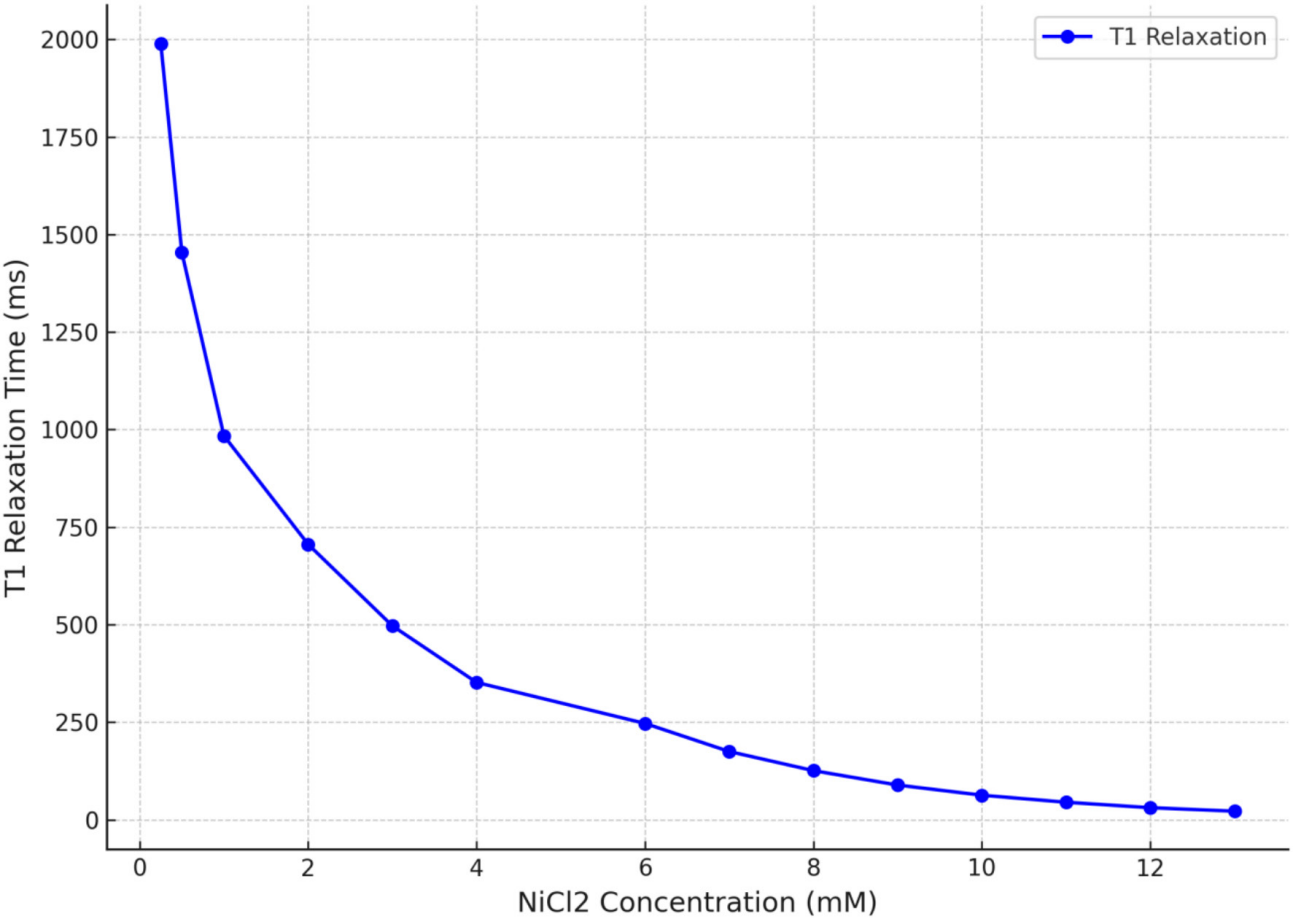
T1 relaxation time as a function of NiCl_2_ concentration, depicted as a curve, ranging from concentrations of 0.25–13 mM and T1 relaxation times of up to 2000 ms, based on phantom measurements. Abbreviations: mM, millimolar; ms, milliseconds; NiCl_2_, nickel (II) chloride.

Initial validation of the MFA sequence was conducted using a NiCl₂ phantom with concentrations ranging from 0.25 to 13 mM (see Table 1). The T1 relaxation times correlated strongly with the NiCl₂ concentrations, displaying consistent, reproducible values across different concentrations. With increasing NiCl₂ concentration, T1 relaxation times decreased systematically, verifying the sequence's capability to measure T1 values accurately across a broad range (see Figure 3). These results confirm that the MFA sequence provides reliable T1 measurements under controlled conditions, ensuring its suitability for in vivo studies.

**TABLE 1:** Measured T1 relaxation times for NiCl₂ concentrations (0.25–13 mM) during validation of the MFA sequence—Phantom measurements at 1.5 Tesla MRI.

NiCl₂ concentration (mM)	T1 relaxation time (ms)
0.25	1989.0
0.5	1454.0
1	984.1
2	706.0
3	496.7
4	351.5
6	247.1
7	175.3
8	125.9
9	89.0
10	62.7
11	44.5
12	30.8
13	21.7

Abbreviations: mM, millimolar; ms, milliseconds; NiCl₂, nickel (II) chloride.

### 3.2 In Vivo T1 Mapping: MOLLI Versus MFA

In a cohort of 20 healthy volunteers, T1 measurements were performed at rest and under stress using both MOLLI and MFA sequences.

Patient characteristics are summarized in Table 2.

**TABLE 2:** Patient characteristics. Summary of demographic, anthropometric, and clinical data for the study cohort (*n* = 20).

Parameter	Total (± SD)
Number of participants (*n*)	20
Women (*n*, %)	10 (50%)
Age (years)	25.8 (± 2.8)
Height (m)	1.78 (± 0.10)
Weight (kg)	69.5 (± 11)
Body mass index (kg/m^2^)	21.9 (± 2.1)
Resting heart rate (x/min)	70.9 (± 10.8)
Heart rate under stress (x/min)	84.5 (± 10.5)
Heart rate increase (%)	20 (± 13%)
Arterial hypertension (%)	0%
Hypercholesterolemia (%)	0%
Diabetes mellitus (%)	0%
Positive family history of cardiovascular disease (*n*, %)	2 (10%)
Smokers (*n*)/pack‐years	2/2.75 (± 3)

Abbreviations: kg, kilograms; m, meters; *n* = number; SD, standard deviation; x/min, heartbeats per minute.

Baseline T1 relaxation times for MOLLI were 1041 ± 28 ms at rest and 1058 ± 34 ms under stress, demonstrating a slight but significant increase with heart rate elevation (*p* < 0.05; see Table 3). In contrast, the MFA sequence showed T1 values of 1069 ± 160 ms at rest and 1035 ± 145 ms under stress, with no significant change observed between conditions (*p* > 0.05; see Table 3). This demonstrates the MFA is frequency independent, providing stable T1 values under varied physiological conditions.

**TABLE 3:** Descriptive statistics of MOLLI and MFA sequence T1 times at rest, under stress, and overall. The table summarizes the number of measurements (*N*), minimum T1 relaxation times (ms), maximum T1 relaxation times (ms), mean T1 relaxation times (ms), and standard deviation (ms) for MOLLI and MFA sequences.

Sequence	Condition	N	T1 relaxation time minimum (ms)	T1 relaxation time maximum (ms)	T1 Relaxation time mean (ms)	Standard deviation (ms)
MOLLI	Rest	20	985	1085	1041	28
MOLLI	Stress	20	1004	1129	1058	34
MFA	Rest	20	860	1332	1069	161
MFA	Stress	20	757	1287	1035	145
MOLLI	Overall	40	985	1129	1050	32
MFA	Overall	40	757	1332	1052	152

Abbreviations: MFA, multiflip angle; MOLLI, modified Look–Locker inversion recovery; ms, milliseconds.

### 3.3 Regional T1 Analysis

**FIGURE 4:**
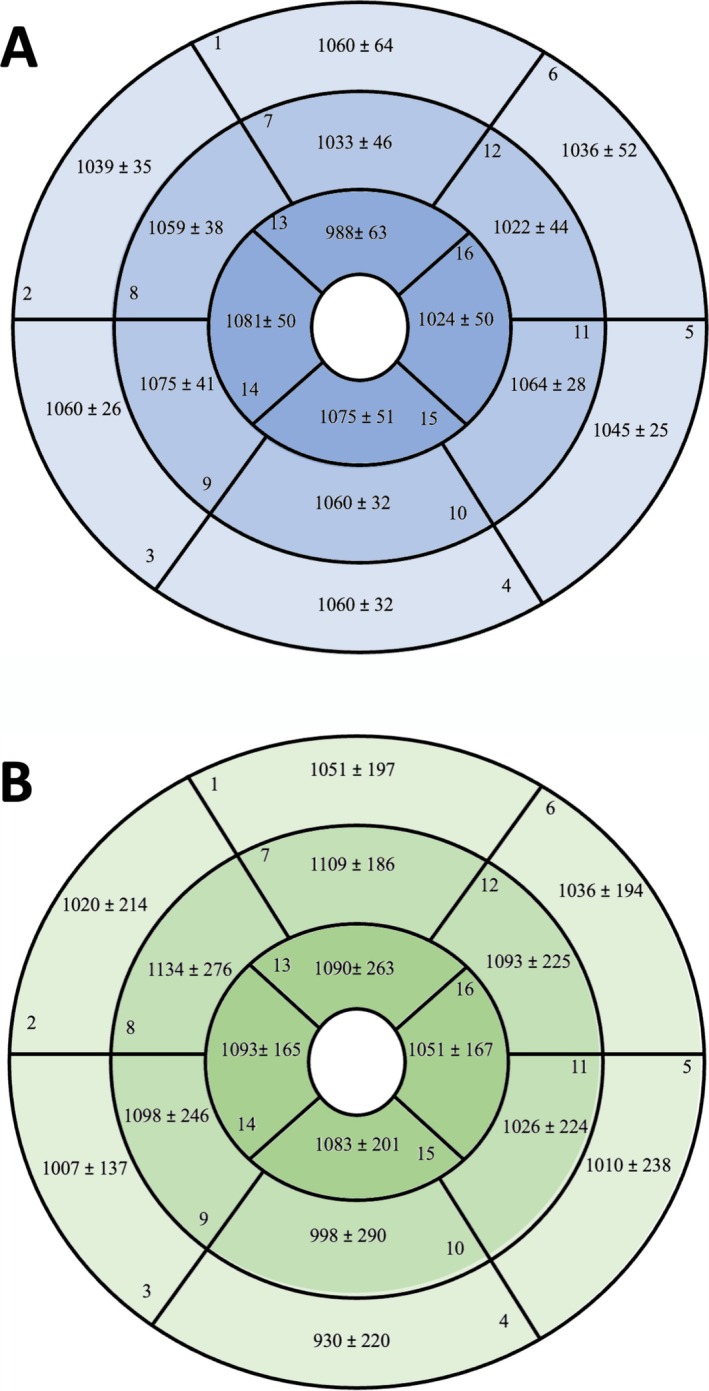
The 16‐segment model of the American Heart Association, presented per segment with mean values and standard deviations. The anatomical orientation of the rings is as follows: The outer ring represents the basal segments, the middle ring the midventricular segments, and the inner ring the apical segments. Mean regional measures ± SD (A) for the MOLLI sequence and (B) for the MFA sequence.

T1 relaxation times were analyzed regionally across the 16‐segment American Heart Association (AHA) model, categorizing segments into basal, midventricular, and apical regions. For the MOLLI sequence, significant differences were observed between the basal (e.g., anterior septal) and lateral segments, with the septal regions generally showing higher T1 times (see Figure 4A). These differences were less pronounced in the MFA sequence (see Figure 4B), which maintained more consistent values across all segments, indicating reduced susceptibility to motion artifacts and local variability. Specifically, the basal inferior segment displayed the largest T1 discrepancy with MOLLI (1060 ± 32 ms vs. 930 ± 220 ms with MFA; *p* < 0.001), suggesting MFA's enhanced robustness for regional mapping (see Table 4).

**TABLE 4:** Average T1 relaxation times per segment measured with MOLLI and MFA sequences. The table lists the basal segments (1–6), midventricular segments (7–12), and apical segments (13–16). Shown are the T1 relaxation times measured with the MOLLI sequence (in milliseconds) with the corresponding standard deviation, compared to the MFA sequence (in milliseconds) with the respective standard deviation. Additionally, the *p* value for significance testing of the differences between the relaxation times measured by both sequences is provided.

No.	Segment	T1 MOLLI (ms) ± SD	T1 MFA (ms) ± SD	*p* value
Basal segments				
1	Basal anterior	1060 ± 64	1051 ± 197	0.848
2	Basal anteroseptal	1039 ± 35	1020 ± 214	0.581
3	Basal inferoseptal	1060 ± 26	1007 ± 137	0.021
4	Basal inferior	1060 ± 32	930 ± 220	0.001
5	Basal inferolateral	1045 ± 25	1010 ± 238	0.361
6	Basal anterolateral	1036 ± 52	1036 ± 194	1.000
Midventricular segments				
7	Midanterior	1033 ± 46	1109 ± 186	0.016
8	Midanteroseptal	1059 ± 38	1134 ± 276	0.096
9	Midinferoseptal	1075 ± 41	1098 ± 246	0.563
10	Midinferior	1060 ± 32	998 ± 290	0.187
11	Midinferolateral	1064 ± 28	1026 ± 224	0.293
12	Midanterolateral	1022 ± 44	1093 ± 225	0.057
Apical segments				
13	Apical anterior	988 ± 63	1090 ± 263	0.021
14	Apical septal	1081 ± 50	1093 ± 165	0.662
15	Apical inferior	1075 ± 51	1083 ± 201	0.808
16	Apical lateral	1024 ± 50	1051 ± 167	0.332

Abbreviations: MFA, multiflip angle; MOLLI, modified Look–Locker inversion recovery; ms, Millisekunden; SD, standard deviation.

### 3.4 Heart Rate Dependency

**FIGURE 5:**
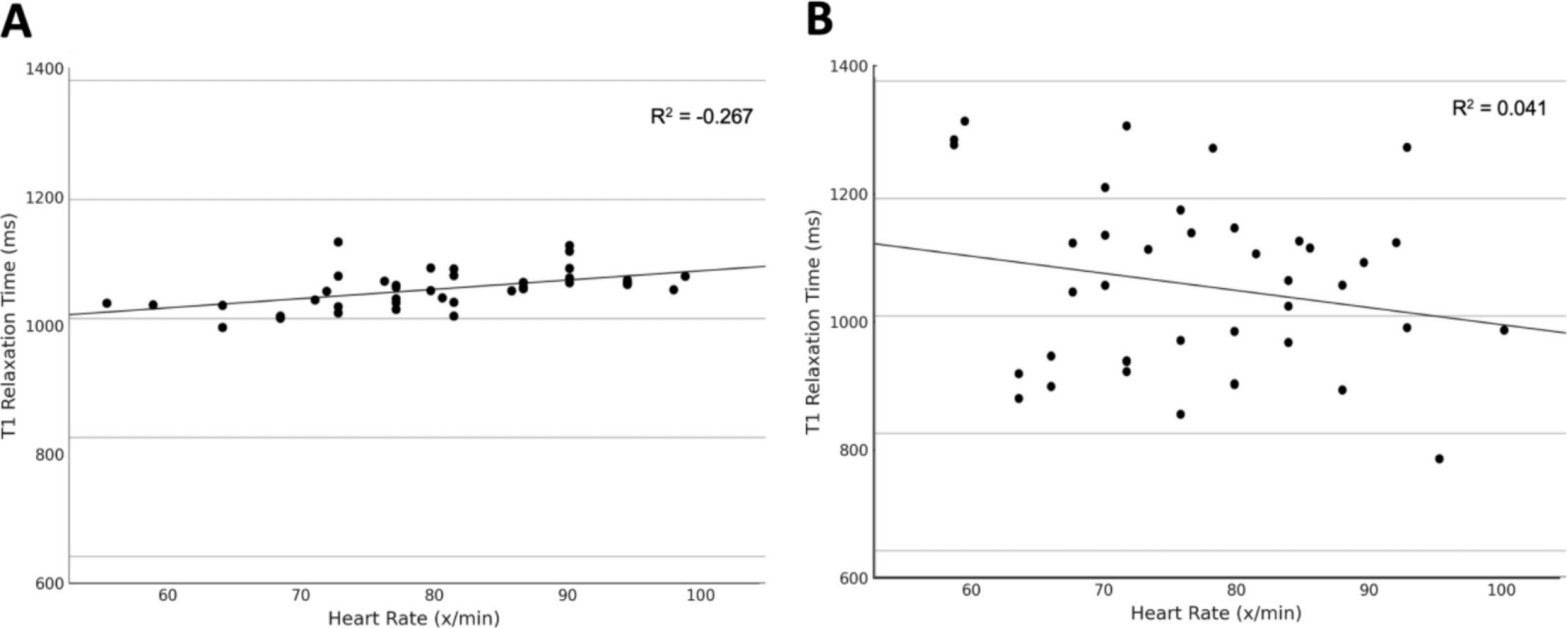
Scatter plot with linear regression analysis. Examining the relationship between participants' heart rate (measured in beats per minute) and the measured T1 relaxation time (measured in milliseconds) for MOLLI sequence in (A) and MFA sequence in (B). Heart rates of the participants range between 50 and 110 beats per minute, whereas T1 relaxation times range between 600 and 1400 ms. Abbreviations: ms, milliseconds; x/min, beats per minute.

The study examined the heart rate dependency of T1 relaxation times in both sequences by correlating T1 values with heart rates, measured at rest and under stress. For MOLLI, a moderate positive correlation (*R* = 0.52, *p* < 0.001) was noted, with T1 values increasing alongside heart rate (see Figure 5A). Conversely, the MFA sequence exhibited no significant correlation with heart rate (*R* = −0.2, *p* = 0.209), confirming its frequency independence (see Figure 5B).

### 3.5 Intraobserver and Interobserver Variability

**FIGURE 6:**
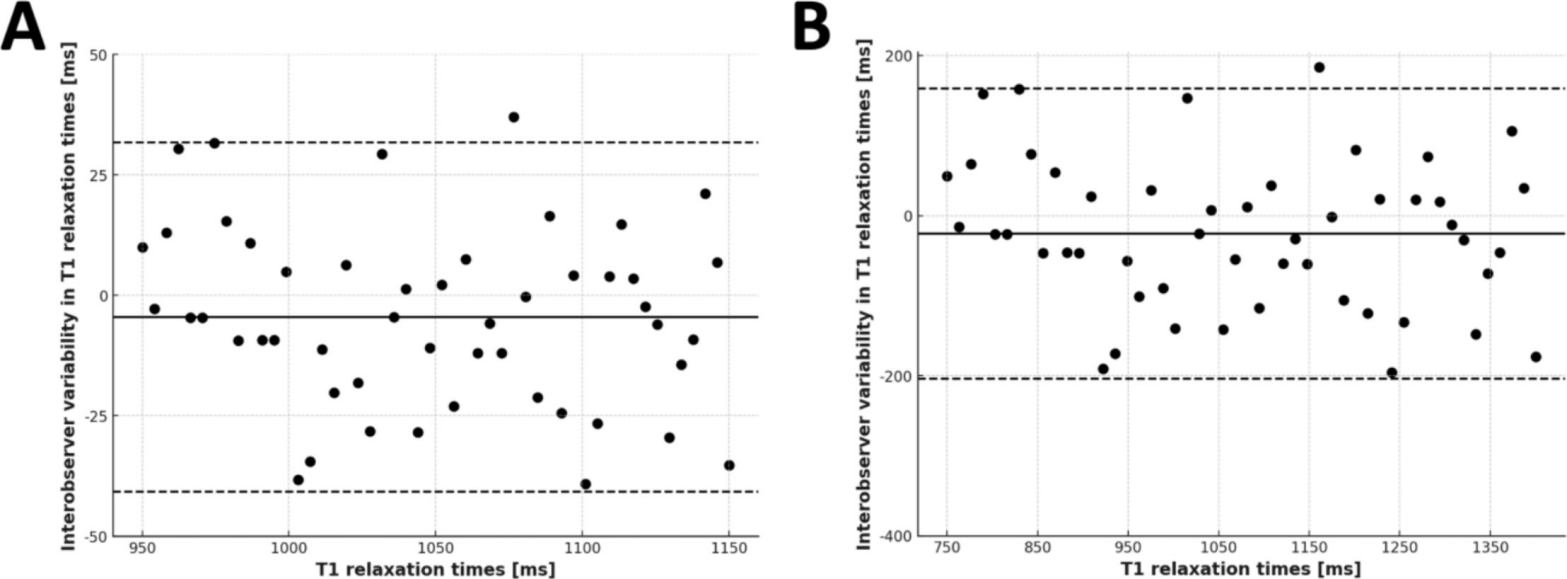
Interobserver variability analysis of T1 relaxation times. (A) Bland–Altman plot illustrating the interobserver variability of T1 relaxation time measurements using the MOLLI sequence. The difference between observers (in milliseconds) is plotted against the mean T1 values. (B) Corresponding Bland–Altman plot for the MFA sequence, depicting interobserver variability in T1 relaxation time measurements. Mean differences and limits of agreement are indicated in milliseconds. Abbreviations: MFA, multiflip angle; MOLLI, modified Look–Locker inversion recovery; ms, milliseconds.

The study assessed intraobserver and interobserver variability using Bland–Altman plots for both sequences (see Figure 6). MOLLI measurements showed a mean difference of −9.23 ms with a variation coefficient of 1.4% (Figure 6A), indicating moderate agreement between observers. In contrast, MFA yielded a mean difference of 12 ms with a variation coefficient of 5.6% (Figure 6B) and wider limits of agreement due to higher absolute values. Despite this, MFA's interobserver consistency was within acceptable limits for clinical applications. The intraobserver variability analysis demonstrated a mean difference in T1 relaxation times between repeated measurements of 9.2 ms (± 20.6 ms) with a correlation coefficient of *R* = 0.90, reflecting strong measurement stability. The interobserver variability analysis showed a mean difference in T1 relaxation times between the two observers of 11.7 ms (± 114 ms), with a correlation coefficient of *R* = 0.86, indicating a high level of reproducibility. These findings suggest that although MFA exhibits slightly higher interobserver variability compared to MOLLI, its reliability remains within clinically acceptable thresholds.

### 3.6 Subgroup Analysis by Heart Rate Increase

To explore the impact of varying heart rate responses, participants were divided into three groups based on heart rate increase from rest to stress: low (< 15%), moderate (15%–25%), and high (> 25%) increases (see Table 5). Across these groups, no significant differences in T1 relaxation times were observed with the MFA sequence, whereas MOLLI showed a consistent trend toward higher T1 times with greater heart rate increases. This subgroup analysis supports MFA's robustness across a spectrum of physiological responses, reinforcing its potential utility in diverse patient populations.

**TABLE 5:** Subgroup characteristics and T1 relaxation times after dividing the cohort into three groups based on heart rate change. The table presents subgroup characteristics and T1 relaxation times for the cohort divided into three groups according to the above‐described heart rate change. Parameters include heart rate increase (percentage), number of participants, gender distribution, age, height, weight, BMI, resting heart rate, heart rate under stress, and T1 relaxation times measured with MOLLI and MFA sequences (in milliseconds), along with their respective standard deviations and *p* values for statistical significance.

No.	Segment	T1 MOLLI (ms) ± SD	T1 MFA (ms) ± SD	*p* value
Basal segments				
1	Basal anterior	1060 ± 64	1051 ± 197	0.848
2	Basal anteroseptal	1039 ± 35	1020 ± 214	0.581
3	Basal inferoseptal	1060 ± 26	1007 ± 137	0.021
4	Basal inferior	1060 ± 32	930 ± 220	0.001
5	Basal inferolateral	1045 ± 25	1010 ± 238	0.361
6	Basal anterolateral	1036 ± 52	1036 ± 194	1.000
Midventricular segments				
7	Midanterior	1033 ± 46	1109 ± 186	0.016
8	Midanteroseptal	1059 ± 38	1134 ± 276	0.096
9	Midinferoseptal	1075 ± 41	1098 ± 246	0.563
10	Midinferior	1060 ± 32	998 ± 290	0.187
11	Midinferolateral	1064 ± 28	1026 ± 224	0.293
12	Midanterolateral	1022 ± 44	1093 ± 225	0.057
Apical segments				
13	Apical anterior	988 ± 63	1090 ± 263	0.021
14	Apical septal	1081 ± 50	1093 ± 165	0.662
15	Apical inferior	1075 ± 51	1083 ± 201	0.808
16	Apical lateral	1024 ± 50	1051 ± 167	0.332

Abbreviations: kg, kilogram; m, meter; MFA, multiflip angle; MOLLI, modified Look–Locker inversion recovery; ms, milliseconds; *n*, number; x/min = heartbeats per minute.

### 3.7 Sex‐Related Differences

**FIGURE 7:**
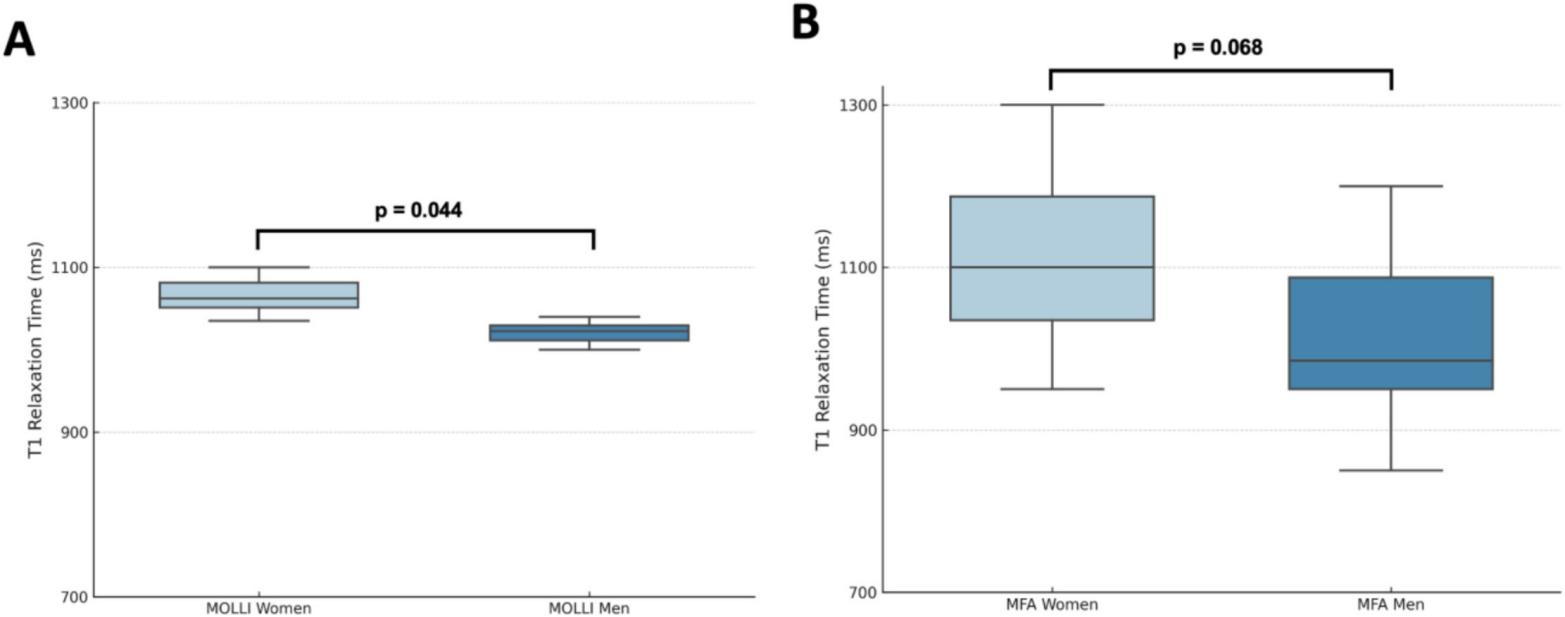
T1 relaxation time values for women and men compared using box plots. (A) MOLLI sequence and (B) MFA sequence. The plots display mean values and measurement ranges. T1 relaxation times are measured in milliseconds, within a possible range of 700 to 1300 ms. Abbreviations: MFA, multiflip angle; MOLLI, modified Look–Locker inversion recovery; ms, milliseconds.

A comparison of T1 relaxation times between sexes revealed higher values in females for both MOLLI and MFA sequences. For MOLLI, the average T1 relaxation time was 1064 ± 32 ms in females versus 1035 ± 25 ms in males, a statistically significant 3% difference (*p* = 0.044) (see Figure 7A). The MFA sequence similarly showed higher T1 values in females (1113 ± 132 ms) compared to males (991 ± 148 ms) (see Figure 7B). However, this difference did not reach statistical significance (*p* = 0.068). Although both sequences exhibit sex‐dependent trends, the MFA sequence appears less influenced by gender‐specific variability.

### 3.8 Age‐Related Differences

The analysis of age‐related effects on T1 relaxation times showed no statistically significant correlation for either sequence. The study cohort, aged 22–31 years (mean: 25.8 ± 2.8 years), exhibited a slight negative trend between age and T1 values, with correlation coefficients of *R* = −0.22 (*p* = 0.17) for MOLLI and *R* = −0.28 (*p* = 0.08) for MFA.

## 4 Discussion

This study aimed to evaluate the newly developed MFA sequence in myocardial T1 mapping, with a focus on overcoming the limitations of the widely used MOLLI sequence. The primary objectives were to test the frequency independence and stability of MFA compared to MOLLI, particularly under conditions of varying heart rates and motion. The results indicate that the MFA sequence demonstrates significant advantages in terms of heart rate and motion independence, while offering stable T1 values with reduced variability, thus supporting its potential application in clinical and research settings.

### 4.1 Validation and Reliability of the MFA Sequence

The phantom‐based validation of the MFA sequence demonstrated strong reliability in T1 measurements across various concentrations of NiCl₂, with T1 values highly consistent with established standards [21, 25]. Notably, the stability of the MFA sequence across a broad range of T1 values, even in controlled settings, is indicative of its precision and reproducibility, which are essential qualities for clinical applications.

### 4.2 Comparison of MFA and MOLLI: Frequency Independence

A key finding of this study is the demonstrated frequency independence of the MFA sequence, a significant improvement over MOLLI. Although MOLLI displayed a moderate positive correlation between heart rate and T1 values (*R* = 0.52, *p* < 0.001), the MFA sequence showed no significant correlation (*R* = −0.2, *p* = 0.209), confirming its robustness in high–heart rate conditions and potential suitability for patients with arrhythmias [26]. This heart rate independence aligns with recent findings that heart rate variations can lead to measurement inaccuracies in MOLLI due to incomplete recovery of longitudinal magnetization between heartbeats [5]. The MFA sequence, by contrast, achieves a stable steady‐state image and minimizes the effects of cardiac motion, thereby reducing artifacts and maintaining accuracy across variable heart rates [13].

### 4.3 Stability and Variability in T1 Measurements

The intraindividual T1 variability analysis further underscores the advantages of the MFA sequence. In this study, T1 measurements taken with MOLLI exhibited higher variability under stress conditions compared to those taken at rest, reflecting MOLLI's sensitivity to physiological changes. In contrast, MFA showed no significant difference in T1 values between rest and stress, indicating a higher level of measurement stability. This consistent performance is particularly relevant for patients with fluctuating physiological states, such as those undergoing stress testing or experiencing arrhythmias. The reduced standard deviation observed in MFA measurements suggests a lower degree of result scatter, enhancing the precision and reliability of T1 assessments in both clinical and experimental settings [27].

### 4.4 Regional and Segmental Analysis of T1 Values

The regional analysis of T1 values across myocardial segments revealed that MOLLI measurements varied significantly between basal and lateral segments, whereas the MFA sequence showed more consistent values across all segments. For instance, the basal inferior segment displayed notably higher T1 values in MOLLI than in MFA, suggesting that MFA is less susceptible to regional artifacts often induced by motion or anatomical differences. The uniformity of MFA‐derived T1 values across regions indicates that this sequence might be better suited for assessing global myocardial health, with minimized segmental bias, which is often a challenge in MOLLI‐based imaging [28]. This is especially advantageous in clinical cases where precise regional T1 values are critical for diagnosing localized myocardial abnormalities [29].

### 4.5 Intraobserver and Interobserver Variability

The interobserver variability analysis revealed a lower degree of consistency in MFA (0.86) measurements compared to MOLLI (0.90), as reflected in the Bland–Altman plots. The ICC for MOLLI was slightly higher than for MFA, indicating better observer agreement for MOLLI. However, this may be attributed to MOLLI's heart rate dependency, which could provide an artificial boost to consistency by producing more uniform values under stable heart rate conditions [30]. The higher variability observed in MFA may be due to its broad applicability across heart rates and motion conditions, suggesting that further refinement in MFA protocols could enhance reproducibility in clinical settings [28].

### 4.6 Sex and Age Effects

In this study, female participants exhibited a tendency toward higher T1 values than male participants with the MOLLI sequence (MOLLI: 1064 ± 32 ms vs. MFA: 1035 ± 25 ms; *p* = 0,044), consistent with previous literature that associates higher T1 values with female sex [31]. However, the MFA sequence did not show a significant difference in T1 values between sexes (MOLLI: *R* = − 0.22; *p* = 0.17 vs. MFA: *R* = − 0.28; *p* = 0.08), indicating that it may offer more reliable measurements that are less affected by biological variability. Additionally, the study found no correlation between age and T1 relaxation times with either sequence, which aligns with recent findings suggesting that age‐related changes in myocardial T1 are not significant in healthy populations [32]. These findings underscore the potential of MFA for providing stable T1 values across demographic variables, a promising feature for broad clinical use.

### 4.7 Limitations

This study has certain limitations. The sample size, although adequate for detecting statistical significance in primary endpoints, may limit the generalizability of subgroup analyses. Nevertheless, similar studies investigating experimental MRI sequences have also utilized small sample sizes of healthy volunteers, making our study design consistent with established research in this field [4, 10]. Additionally, the study was conducted in a single center, which may introduce biases associated with site‐specific equipment or imaging protocols. Future studies with larger and more diverse populations, as well as multicenter trials, are warranted to validate these findings across different clinical settings and scanner models. Furthermore, although the MFA sequence demonstrated reduced heart rate dependency, further optimization may be needed to address the slightly higher interobserver variability observed compared to MOLLI [33]. Although our study effectively demonstrates the motion robustness and heart rate independence of MFA, its validation in a clinically relevant patient population remains an important next step. The decision to initially focus on healthy subjects was made to ensure a controlled experimental setting, minimizing confounding factors introduced by myocardial pathologies. Given the small sample size of 20 participants, the inclusion of patients with arrhythmias could have led to increased variability and potential bias, limiting the statistical power to derive meaningful conclusions. However, we acknowledge that assessing the performance of MFA in pathological myocardial states is crucial for its broader clinical applicability. Future studies are already planned to investigate its robustness in patients with cardiac arrhythmias and under stress conditions, further substantiating its diagnostic value in real‐world clinical scenarios.

## 5 Conclusions

In summary, this study demonstrates that the MFA sequence provides a robust, frequency‐independent alternative to MOLLI for myocardial T1 mapping. The frequency independence of MFA makes it a valuable tool for patients with arrhythmias or those undergoing stress testing, where heart rate fluctuations could affect T1 accuracy with MOLLI. Although MOLLI showed lower interobserver variability, the advantages of MFA in heart rate and motion independence suggest it holds promise for more accurate and reliable T1 mapping in complex clinical scenarios. The findings support the integration of MFA into clinical practice for reliable T1 mapping, with potential to improve diagnostic accuracy and patient outcomes in cardiac imaging. Further research, focusing on standardizing MFA protocols and expanding its use in diverse clinical settings, will help solidify its role in cardiac imaging.

## Author Contributions

SH was responsible for conception of study design, data acquisition and analysis, statistical assessment, and writing of the manuscript. MR and VK involved in analysis, writing, in drafting, and final approval of the manuscript submitted. VK and CS involved in data acquisition. TV and DL involved in interpretation of the data and critical review of the manuscript. MO and SM involved in analysis and interpretation of data. All authors read and approved the final manuscript.

## Ethics Statement

The analysis of deidentified patient data was approved by the local institution's ethics committee. Documentation of consent was waived for this retrospective study.

## Conflicts of Interest

The authors declare no conflicts of interest.

## Consent

Documentation of consent was waived for this prospective study, which utilized nonblinded patient data.

## Data Availability

The datasets used and/or analyzed during the current study are available from the corresponding author on reasonable request.
